# Correction to: Disruption of endothelial adherens junctions by high glucose is mediated by protein kinase C-β–dependent vascular endothelial cadherin tyrosine phosphorylation

**DOI:** 10.1186/s12933-017-0614-7

**Published:** 2017-10-20

**Authors:** Mehran Haidari, Wei Zhang, James T. Willerson, Richard A. F. Dixon

**Affiliations:** 1 Department of Internal Medicine, Division of Cardiology, The University of Texas Medical School at Houston, Houston, TX 77030 USA; 2 Texas Heart Institute at St. Luke’s Episcopal Hospital, PO Box 20345 C1000, Houston, TX 77030 USA

## Correction to: Cardiovasc Diabetol (2014) 13:112 DOI 10.1186/1475-2840-13-112

This article was unintentionally published twice in this journal, by the same authors. The following should be considered the version of record and used for citation purposes: “Mehran Haidari, Wei Zhang, James T Willerson and Richard AF Dixon, Disruption of endothelial adherens junctions by high glucose is mediated by protein kinase C-β–dependent vascular endothelial cadherin tyrosine phosphorylation, Cardiovascular Diabetology, Volume 13, Issue 1, doi: 10.1186/1475-2840-13-112“. The duplicate “Mehran Haidari, Wei Zhang, James T Willerson and Richard AF Dixon, Disruption of endothelial adherens junctions by high glucose is mediated by protein kinase C-β–dependent vascular endothelial cadherin tyrosine phosphorylation, Cardiovascular Diabetology, Volume 13, Issue 1, doi: 10.1186/1475-2840-13-105“ is to be ignored. The Publisher apologizes to the readers of the journal for not detecting the duplication during the publication process.
